# Repetitive Transcranial Magnetic Stimulation Attenuates the Perception of Force Output Production in Non-Exercised Hand Muscles after Unilateral Exercise

**DOI:** 10.1371/journal.pone.0080202

**Published:** 2013-11-22

**Authors:** Stuart Goodall, Alan St Clair Gibson, Bernhard Voller, Mike Lomarev, Glyn Howatson, Nguyet Dang, Tibor Hortobágyi, Mark Hallett

**Affiliations:** 1 Faculty of Health and Life Sciences, Northumbria University, Newcastle upon Tyne, United Kingdom; 2 Department for Neurology, Medical University of Vienna, Vienna, Austria; 3 Bekhterev Neuropsychological Institute, St. Petersburg, Russian Federation; 4 Water Research Group, School of Environmental Sciences and Development, Northwest University, Potchefstroom, South Africa; 5 Human Motor Control Section, National Institute of Neurological Disorders and Stroke, National Institutes of Health, Bethesda, Maryland, United States of America; 6 University of Groningen Medical Center, Groningen, The Netherlands; University of California, Merced, United States of America

## Abstract

We examined whether unilateral exercise creates perception bias in the non-exercised limb and ascertained whether rTMS applied to the primary motor cortex (M1) interferes with this perception. All participants completed 4 interventions: 1) 15-min learning period of intermittent isometric contractions at 35% MVC with the trained hand (EX), 2) 15-min learning period of intermittent isometric contractions at 35% MVC with the trained hand whilst receiving rTMS over the contralateral M1 (rTMS+EX); 3) 15-min of rTMS over the ‘trained’ M1 (rTMS) and 4) 15-min rest (Rest). Pre and post-interventions, the error of force output production, the perception of effort (RPE), motor evoked potentials (MEPs) and compound muscle action potentials (CMAPs) were measured in both hands. EX did not alter the error of force output production in the trained hand (Δ3%; P>0.05); however, the error of force output production was reduced in the untrained hand (Δ12%; P<0.05). rTMS+EX and rTMS alone did not show an attenuation in the error of force output production in either hand. EX increased RPE in the trained hand (9.1±0.5 vs. 11.3±0.7; P<0.01) but not the untrained hand (8.8±0.6 vs. 9.2±0.6; P>0.05). RPE was significantly higher after rTMS+EX in the trained hand (9.2±0.5 vs. 10.7±0.7; P<0.01) but ratings were unchanged in the untrained hand (8.5±0.6 vs. 9.2±0.5; P>0.05). The novel finding was that exercise alone reduced the error in force output production by over a third in the untrained hand. Further, when exercise was combined with rTMS the transfer of force perception was attenuated. These data suggest that the contralateral M1 of the trained hand might, in part, play an essential role for the transfer of force perception to the untrained hand.

## Introduction

A body of evidence exists demonstrating that the primary motor cortex (M1), as part of a network of brain regions, contributes to the generation of force output and the retention of motor skills [1]–[3]. Transcranial magnetic stimulation (TMS) is a method that can be used to investigate changes in motor cortical function [4]–[6]. Moreover, it has been suggested that ‘*effort*’ [7] or the ‘*sense of effort*’ [8] results from a corollary discharge associated with motor cortical efferent activity [9]. It is unclear however, just how the ‘*sense of effort’* may affect the motor programs or neural networks responsible for the generation and retention of a desired force output to occur.

Studies using human and animal models have demonstrated that learnt ability acquired with one hand transfers through to the other hand, a process known as ‘intermanual transfer’ [10], [11] or ‘cross education’ [12]–[14]. The neural networks responsible for such transfer of learning, however, are not well understood. Functional imaging work demonstrates that the primary M1 contralateral to the untrained hand is active during motor sequence learning tasks [11], [15]. Thus, the transfer of learning may arise via inter-hemispheric links through the M1 [16]. Recently, a number of studies investigating the transfer of learning after ballistic finger movements [14], [17]–[19], have shown that forceful tasks induce neural adaptations (twitch forces evoked via TMS) similar to those that are thought to mediate the response to strength training which can occur within a single session (<1 h) [20]. Collectively, it is apparent that changes within M1 play a crucial role in mediating the responses in both trained and untrained limbs following unilateral training. However, the effect that rTMS delivered, *during* unilateral training, has on the associated transfer of learning and perception of force output to an untrained limb is unknown.

Pinch force control is a model that has been used to investigate the transfer of learning [21]. Moreover, pinch force control relies on the activation of an extensive cortical network and requires integrity of the corticospinal tract, thus engaging more neuronal resources than gross whole-hand pinch control [21]. Precise pinch force requires fine force control and is important for carrying out daily living activities and loss of this ability is present after brain lesions like stroke [22]. One method known to disrupt activity in specific cortical regions [23] and diminish exercise induced gains [24], is repetitive TMS (rTMS). Specifically, low frequency rTMS (1 Hz) induces inhibitory effects on motor cortical excitability through the generation of a temporary ‘virtual lesion’ [25]. Muellbacher et al. [26] showed that when *learning* a new task, such as pinch force accuracy, the newly acquired motor skill is consolidated in M1. Furthermore, Voss et al. [27] found that reducing excitability of M1, using theta burst rTMS, improved participants’ force matching ability. The authors attributed the findings to a reduction in sensory attenuation via a divergence between the efferent copy of information generated and the motor output produced [27]. In an extension of this work, Therrien et al. [28] showed that a reduced M1 excitability, induced by theta burst rTMS, reduced the common overproduction in force following the removal of visual feedback. These authors also attributed their findings to the disruption of sensory attenuation processes and differences between predicted and actual afferent information [28]. Given the aforementioned evidence and that numerous studies have reported rTMS protocols induce activity in the contralateral M1, hence influencing excitability of ipsilateral fibres of the corticospinal tract [29]–[32], it is of interest to understand the effect rTMS has on the force output of a limb ipsilateral to the site of stimulation [28].

Another parameter that is known to influence the perception of a desired force output is the sensation of fatigue. A common model to examine the relationship between the sensation of fatigue and perception of force output utilises the contralateral limb-matching method [33], [34]. In this model, participants are required to generate a specified level of force by contracting the muscle of a reference limb to match the subjective magnitude of the force output during fatiguing contractions in the contralateral experimental limb. When participants are required to reproduce the force applied during sustained fatiguing contractions, by generating brief matching contractions in the reference limb, there is a linear increase in the perceived magnitude of the reference force [35], [36]. These findings suggest that force output is monitored by either afferent sensory input from mechanoreceptors or metaboreceptors in the periphery or by knowledge of increased efferent neural command required to maintain a force output in an exercising and non-exercising limb. However, it is unknown to what extent the increased ‘sense of effort’ (i.e., perceived exertion), that manifests during exercise, impacts on the transfer of learning and perceived force output.

Thus, the primary aim of this study was to investigate the specific role of M1 in the capacity for bilateral transfer of force perception following a pinch task and to investigate whether such transfer is altered with rTMS. It was hypothesised that learning patterns within the active M1 would be suppressed by rTMS and secondly, the heightened perceived exertion known to occur during exercise would inhibit the ability to accurately predict pinch force in exercised and non-exercised limbs.

## Methods

### Participants

Thirteen adults (10 men, 3 women; mean ± SD age, 40±12 y) volunteered to participate in the study. Participants gave written, informed consent prior to all experimental procedures, which were approved by the Institutional Review Board at the National Institute for Neurological Disorders and Stroke (NINDS). Of the 13 participants, 12 were right handed and 1 was left handed, as identified using the Oldfield handedness questionnaire [37]. The study was performed in accordance with the Declaration of Helsinki.

### Experimental Design

**Figure 1:**
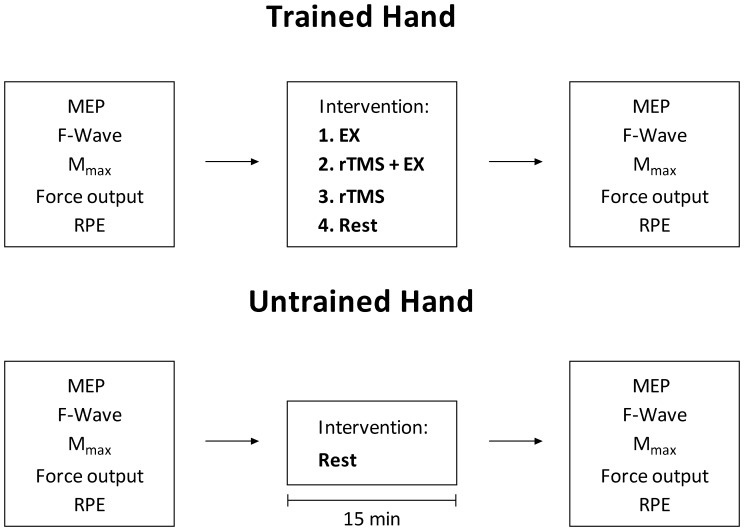
The experimental protocol. Motor evoked potentials (MEP), F-waves, maximal M-waves (M_max_), perceived force error and ratings of perceived exertion (RPE) were recorded before and after each intervention in the trained and untrained hands. The order of interventions was randomised and performed on separate days. Each of the interventions, performed with the ‘trained hand’, lasted for 15 mins and during this time the ‘untrained hand’ remained at rest. Within 5 minutes after each intervention post responses were acquired. EX –15 min of force training with the trained hand; rTMS+EX –15 min of force training and rTMS over M1 region of the trained hand performed concurrently; rTMS –15 min of rTMS over M1 region corresponding to the trained hand; Rest – no intervention.

Figure 1 shows the experimental design; in a randomised, counterbalanced order, each participant underwent the following 4 interventions as separate trials on separate days: 1) 15 min ‘training’ period during which participants performed intermittent (5 s contraction, 5 s rest periods) isometric contractions at 35% maximal voluntary contraction (MVC) with the trained hand (EX); 2) 15 min learning period during which participants performed intermittent (5 s contraction, 5 s rest periods) isometric contractions at 35% MVC with the trained hand whilst receiving rTMS over the contralateral M1 (rTMS+EX); 3) 15 min of rTMS alone over the motor cortex (M1) contralateral to the ‘trained’ hand (rTMS) and 4) 15 min rest with no intervention (Rest). Before and immediately after (within 5 min) each intervention a number of outcome variables were measured in both the trained and the contralateral ‘untrained’ hand. Outcome variables were monitored in the untrained hand at the same time points as the trained hand to assess whether the interventions manipulated effort perception in both hands.

The principal outcome measure was the error in participants’ force output while they estimated 35% of MVC force with their eyes closed during a 5 s contraction. A simple constant error calculation was determined (mean force output in N – the 35% MVC target) and the resulting N value was converted to a percentage of the overall 35% value. For example, in a scenario where a participant’s 35% MVC target is 15 N and their mean pinch force is 20 N they would have overestimated by 5 N (5/15 = 33%); post an intervention the same participant attempts again and their mean force is 16 N, so an overestimation of 1 N (7%) demonstrating an improvement in the accuracy of force output production. Thus, a reduction in the error of force output production in the untrained hand, after the interventions performed with the opposite hand, would demonstrate a transfer of learning. Secondary variables also measured pre and post each intervention included: the rating of perceived exertion (RPE; [38]), motor evoked potentials (MEPs) elicited by single pulse TMS, the maximal motor response (M_max_) and F-waves elicited by peripheral nerve stimulation. RPE was obtained as a measure of the perceived effort and MEPs measured changes in corticospinal excitability with the aim to determine if changes in MEPs were associated with changes in perceived effort. M_max_ and F-waves measured changes in peripheral neuromuscular function.

### Testing Procedures

#### Voluntary Force

Pinch force output of the thumb and index finger was measured using a calibrated load cell (Model 31, Sensotec Inc, Columbus, OH). Prior to all testing the load cell was calibrated across the physiological range by suspending known masses (kg), with regression analysis used to convert raw analogue signals (mV) to force (N). Force output was measured from each participant’s trained (dominant) and untrained (non-dominant) hand. Participants were seated with their elbows and forearms resting on a table in front of them; wrist angle was held constant at anatomical zero; all recordings of pinch force were made from this position. Prior to the initial measurement of maximal voluntary force (MVC), all participants were thoroughly familiarised with the load cell set-up. Before the commencement of experimental procedures in the first intervention, peak MVC was recorded from five attempts (5 s duration) in both hands and used as the MVC for all further subsequent trials. During these trials participants used 35% MVC of their trained hand as the contraction intensity. Force signals were displayed on a computer monitor in front of the participants. When training, participants received continuous visual feedback of force on the computer monitor; visual targets were set at 35% MVC.

#### Electromyography

Electromyographic (EMG) activity of the flexor policis brevis (FPB), flexor digotorum superficialis (FDS) and abductor digiti minimi (ADM) was recorded using silver-silver chloride surface electrodes (inter-electrode distance 2 cm) placed over these muscles in a belly-tendon montage. EMG signals were amplified with a Nicolet Viking electromyography system (gain 1000; Madison, Wisconsin, USA), band-pass filtered (10–2000 Hz), digitised (5 kHz), acquired and later analysed offline. EMG responses were recorded after motor cortex and motor nerve stimulation. EMG was measured in the ADM to monitor potential spread of excitation elicited by rTMS interventions; this measure was used as a warning sign for seizure.

#### Transcranial magnetic stimulation and peripheral nerve stimulation

Single pulse TMS was delivered to the left and right M1 over the optimal scalp position to activate the FPB muscle in the trained and untrained limbs using a figure-8-shaped coil (70 mm diameter) powered by a monophasic magnetic stimulator (Magstim 200, The Magstim Company Ltd. Whitland, UK). The optimal coil positions were marked on the scalp; the intersection of the coil was placed tangentially to the scalp with the handle pointing backwards and laterally at a 45° angle away from the midline over the respective muscles ‘hot spot’. The direction of intracranial current flow within M1 was postero-anterior [39]. Resting motor threshold (rMT) was determined at the beginning of each trial; briefly, TMS was first delivered with the coil placed over the optimal stimulation site at a sub-threshold intensity of 30% maximum stimulator output. Stimulus intensity was then increased in 5% steps until consistent motor evoked potentials (MEPs) with peak-to-peak amplitudes of more than 50 µV were evoked. Thereafter, stimulus intensity was reduced in 1% steps until an intensity was reached that elicited an MEP of at least 50 *μ*V in 5 out of 10 trials [40], [41]. Subsequent stimuli were delivered at 140% rMT and MEP characteristics were determined by averaging 20 single trials pre and post each intervention and expressed relative to the maximal motor response (M-wave; M_max_).

As outlined in the *Experimental Design* section, rTMS was used to assess whether perception of force output and memory formation of force output and fatigue can be attenuated. 1 Hz rTMS at 90% rMT was applied in accordance with appropriate safety recommendations [42]; a figure-8-shaped coil (70 mm diameter) powered by a rapid-rate magnetic stimulator (Magstim Rapid, The Magstim Company Ltd. Whitland, UK) was used to deliver the rTMS. To ensure there was no increase in motor drive resulting from the rTMS beyond that required by the testing protocol, EMG activity was monitored throughout rTMS sessions to ensure increased motor unit firing rate did not occur [42]. Across the experimental sessions rTMS was delivered over M1 contralateral to the trained hand. The coil position used was the optimal position for stimulation of FPB as determined for single pulse TMS. Maximal voluntary force, 35% MVC force levels and resting motor threshold for all intervention groups did not differ (Table 1).

**Table 1:** Maximal voluntary contraction (MVC), 35% MVC force output (35%) and resting motor threshold (rMT) of the flexor policis brevis (FPB) for the trained and untrained hands during all interventions.

	Intervention	MVC (N)	35%	rMT (%)
**Tr. Hand**	Ex	38.2±11.4	13.3±4.0	63.9±9.7
	rTMS/Ex	38.4±9.9	13.5±3.5	63.5±9.7
	rTMS	38.8±11.3	13.6±3.9	66.7±12.3
	Rest	37.8±9.6	13.2±3.3	65.2±9.2
**Ut. Hand**	Ex	34.5±11.2	12.3±4.0	63.2±12.1
	rTMS/Ex	35.6±12.0	12.3±4.0	62.6±11.7
	rTMS	35.2±11.4	12.3±4.0	62.8±11.2
	Rest	34.5±11.2	12.3±4.0	63.1±10.9

Tr. = Trained; Ut. = Untrained. Values are means ± SD.

The ulnar nerve in both limbs was stimulated (1 ms rectangular pulse; model Viking IV, Nicolet Biomedical, Madison, Wisconsin, USA) using bipolar surface electrodes placed at the wrist to determine the M_max_. Supramaximal stimulation was ensured at the beginning of each trial; the intensity of stimulation was increased from a subliminal level until there was no further increase in the M_max_ with increasing stimulation intensity. Once the supramaximal intensity was established five stimuli were delivered pre and post each intervention. In addition to the M_max_, F-waves elicited by the supramaximal stimulus were also measured; these peripheral measures were derived in order to monitor changes in the peripheral neuromuscular system. The properties of evoked potentials measured were the peak-to-peak amplitude and area. The peak-to-peak amplitude was defined as the absolute difference between the maximum and the minimum points (one negative and positive deflection) of the biphasic M-wave [43], V wave [44] or MEP [45]. The area was calculated as the integral of the reflected value of the entire M-wave [43] or MEP [45].

### RPE

Ratings of perceived exertion (RPE) for limb discomfort were obtained using Borg’s 6–20 scale [38]. This scale was constructed for practical use and in measuring increasing levels of perceived exertion. The scale consists of a limited number range from 6 to 20 with verbal descriptors that are anchored to numbers that relate to psycho-physiological perceptions of effort. Specifically, the participants were asked to recall known perceptions of effort to anchor sensations at the top and bottom of the scale. Before and after every intervention, participants were asked to rate the sensation of effort in both the trained and untrained hands.

### Statistical Analyses

All data are presented as means ± SE unless otherwise stated. A 1 way ANOVA was used to determine differences in MVC and rMT and a 3 way ANOVA (Hand [trained vs. untrained;2] × Time [pre vs. post; 2] × Intervention [4]) was used to determine differences in outcome measures (the error in force output production, RPE, MEPs, M_max_ and F-waves) between the trained and untrained hands. A paired samples T-Test was used to reveal differences in EMG activity in the trained and untrained hands during the EX intervention. The level of significance was determined *a priori* (α = 0.05) and *post-hoc* analyses were performed using the least significant difference (LSD) test. All statistical analyses were performed using SPSS (v19, IBM Corporation, New York, USA).

## Results

**Figure 2:**
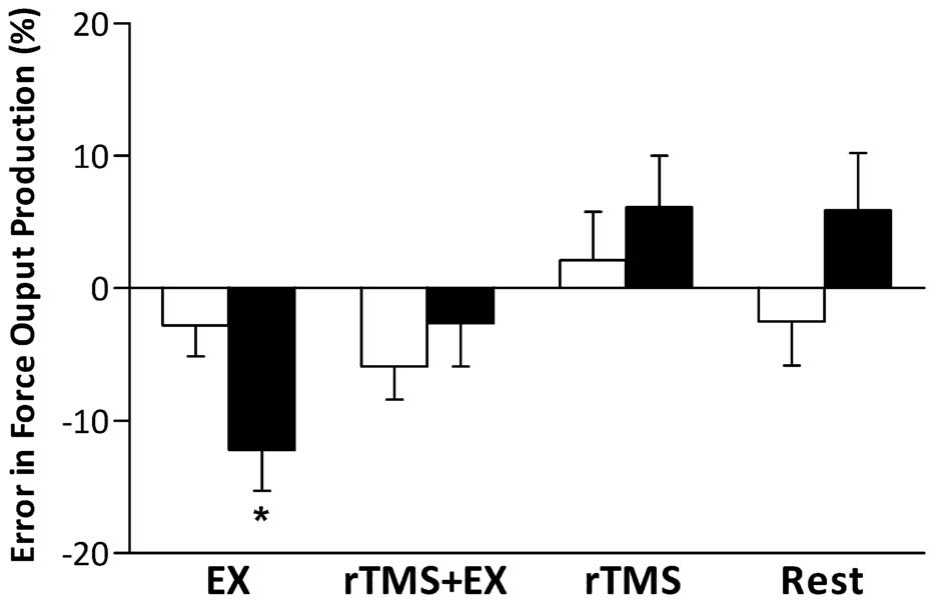
The error in force output production after the fifteen minute interventions (EX –15 min of force training with the trained hand; rTMS+EX –15 min of force training and rTMS over M1 region of the trained hand performed concurrently; rTMS –15 min of rTMS over M1 region corresponding to the trained hand; Rest – no intervention) in both the trained (open bars) and untrained (closed bars) hands. Data are means ± SE for 13 participants. * - P<0.05 trained vs. untrained hand.

MVC and rMT did not differ between the trained and un-trained hands (P>0.05, Table 1). Figure 2 shows the change in the error in participants’ force output after each intervention; there were no differences between baseline values in each intervention. There was no significant main effect between the trained and untrained hand (F_1,12_ = 4.5, P = 0.055) or intervention (F_3,36_ = 1.2, P = 0.341); however, there were significant interactions (time × intervention; F_3,36_ = 3.4, P = 0.029 and hand × time × intervention; F_3,36_ = 3.1, P = 0.038) for the change in the error of force output production. *Post-hoc* comparisons revealed that EX did not alter the error in force output production in the trained hand (15±3 vs. 12±2%; P = 0.611); however, the error in force output production was reduced in the untrained (25±4 vs. 13±3%; P = 0.023), demonstrating a transfer of force perception. rTMS+EXx did not alter the error in force output production in either hand. Similarly, rTMS and rest alone did not affect the error in force output production in the trained or untrained hands.

**Figure 3:**
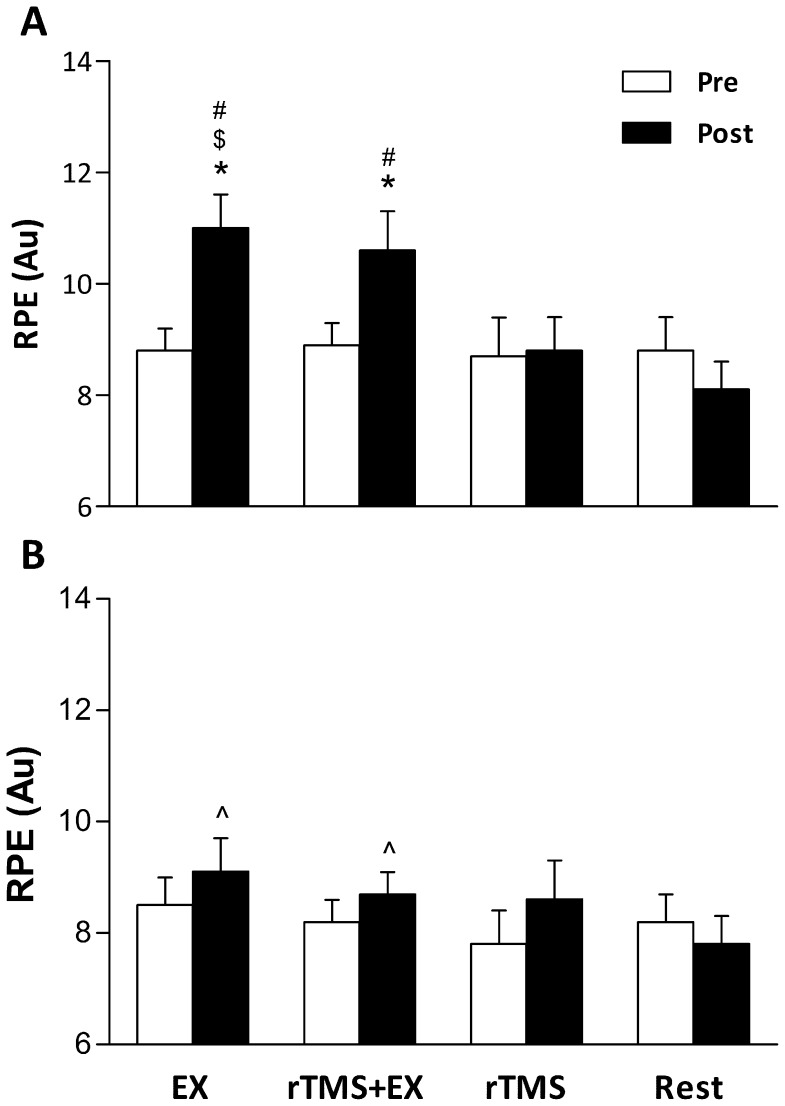
Changes in ratings of perceived exertion (RPE) in the trained (A) and untrained (B) hand before and after the 15 min interventions (EX –15 min of force training with the trained hand; rTMS+Ex –15 min of force training and rTMS over M1 region of the trained hand performed concurrently; rTMS –15 min of rTMS over M1 region corresponding to the trained hand; Rest – no intervention). Data are means ± SE for 13 participants. * - P<0.05 pre vs. post; $ - P<0.05 vs. rTMS post; # - P<0.01 vs. Rest post; ^∧^ - P<0.05 vs. trained hand at the same time point.

There were significant main effects for RPE on hand, intervention and time (P≤0.027). There was a significant interaction (time × intervention; F_3,36_ = 7.2, P = 0.001 and hand × time × intervention; F_3,36_ = 5.6, P = 0.003) for changes in RPE. *Post-hoc* comparisons revealed that EX increased RPE in the trained hand (9.1±0.5 vs. 11.3±0.7; P = 0.018) but not the untrained hand (8.8±0.6 vs. 9.2±0.6; P = 0.622). RPE was also increased in the trained hand during rTMS+EX (9.2±0.5 vs. 10.7±0.7; P = 0.028), but ratings were unchanged in the untrained hand (8.5±0.5 vs. 8.9±0.5; P = 0.565). RPE did not differ in either hand pre to post rTMS or a period of 15 min rest (Figure 3).

**Figure 4:**
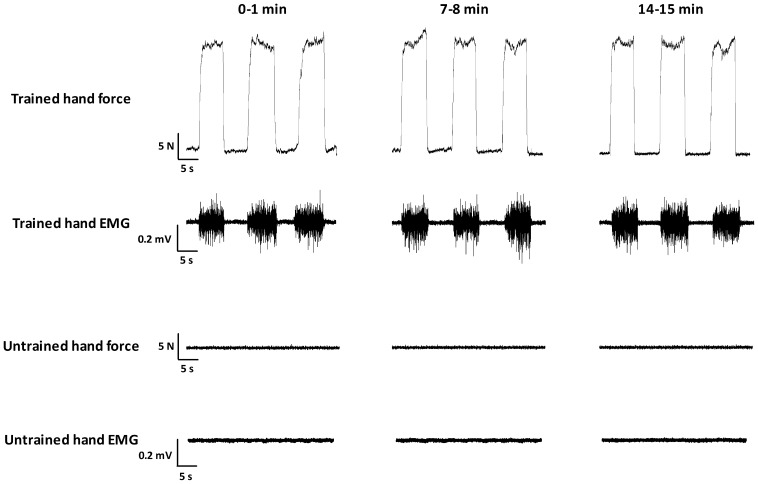
Raw force and EMG traces while a performed the EX intervention. EMG activity shown was recorded from the FPB muscle. Note, during the 35% contractions with the trained hand, no EMG activity was recorded in the untrained hand.

**Figure 5:**
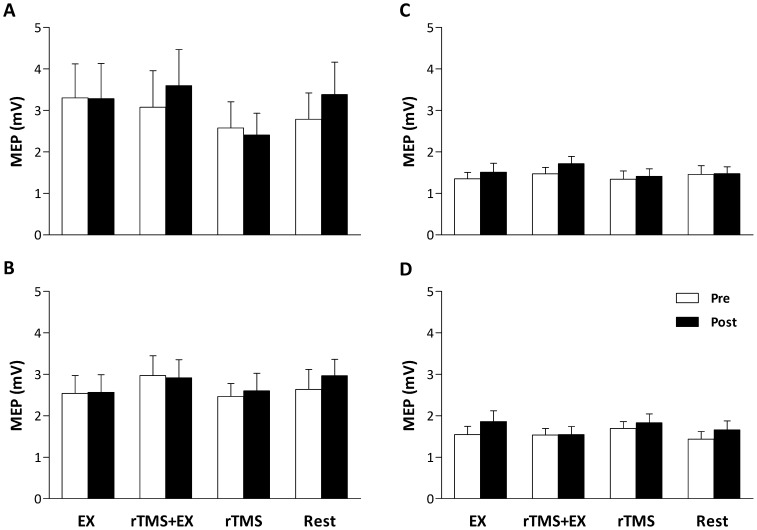
Motor evoked potentials (MEP) evoked in the flexor pollicis brevis (FPB; A and B) and flexor digitorum superficialis (FDS; C and D) muscles of the trained (A and C) and untrained hand (B and D) before and after the 15 min interventions. Data are means ± SE.

**Figure 6:**
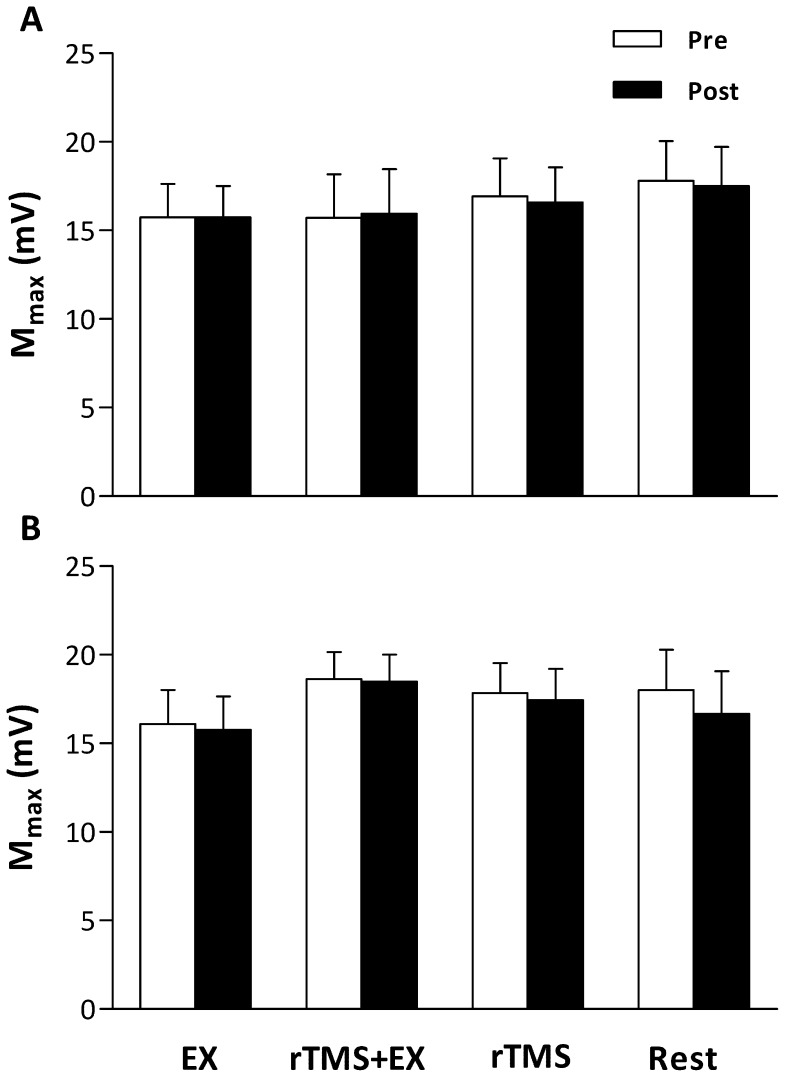
Maximal M-waves (M_max_) evoked in the flexor pollicis brevis (FPB) of the trained (A) and untrained (B) hands before and after the 15 min interventions. Data are means ± SE.

EMG activity of the untrained hand was monitored online during all experiments and recorded, as a control from one participant. From the representative trace (Figure 4), while the participant performed the EX intervention, EMG activity is well modulated to the level of force required in the trained hand, whereas EMG activity in the untrained hand was absent (P<0.01 vs. the trained hand at each time point shown). Despite differences occurring in the error of participants force output between the trained and untrained hands, none of the interventions elicited changes in the response to motor cortex (MEPs [Figure 5] and rMT; main effects = P>0.05) or motor nerve (M_max_ [Figure 6] and F-Waves; main effects = P>0.05) stimulation.

## Discussion

The primary aim of the present study was to determine whether unilateral exercise creates perception bias in the non-exercised limb. We also examined whether rTMS could be used to suppress M1 activity and subsequently alter such perception. The novel finding was that exercise alone reduced the error in force output production by more than a third in the untrained hand; however, when the exercise was combined with rTMS the transfer of reduced error, between hemispheres, did not occur. The observed changes in the error in force output production manipulated by rTMS occurred independent to the perception of effort. Hence, our data suggest that the mechanism responsible for the increased perception of force output in an untrained limb involves the primary motor cortex controlling the trained limb and not the sensory perception of effort.

### Error of Force Output Production and the Transfer of Learning

Evidence suggests that the M1 is a part of a network of brain regions involved during motor learning; further, research using TMS demonstrates that improvements in ballistic motor performance are particularly dependent on modifications within M1 [17], [26]. Muellbacher et al. [26] and Baraduc et al. [46] reported that rTMS over the contralateral motor cortex of the trained limb disrupts the early retention of a newly acquired motor skill. Furthermore, recent evidence from Lee et al. [14] showed that early retention of ballistic performance improvements are impaired by rTMS delivered to the contralateral (trained) hemisphere shortly after practice. Moreover, the transfer of learning is *only* disrupted if rTMS is delivered to the ‘untrained’ hemisphere. This suggests that processes in the *untrained* M1 (rather than the trained M1) contribute to the early retention of ballistic performance improvements in the untrained limb [14]. Our data demonstrate that rTMS delivered to the ‘trained’ M1 *during* exercise disrupts the ability to accurately perceive force output in the untrained limb, whereas if the same exercise is performed without rTMS, the transfer of learning is evident and the perception of force output is improved (Figure 2). This suggests that a transfer of learning is evident *during* motor practice and the mechanism seems to involve the ‘trained’ M1; results which are in line with the ‘bilateral access’ hypothesis [14], [47]. Briefly, the bilateral access hypothesis is based on the observation that performance of a unilateral task will produce motor engrams in the ‘trained’ hemisphere, which via the corpus callosum, can then be accessed by the opposite hemisphere to facilitate task performance [14]. Importantly, being able to judge the level of force produced is the result of 1) discharge of Ib afferents and 2) from an internal neural correlate or copy of the motor command (corollary discharges) sent to the motor neuron pool in the spinal cord [48]. Presumably, this signal is transmitted to the sensory centers in the brain and might possibly reflect the magnitude of the descending motor signal. Due to the unchanged RPE, we suppose that Ib afferents in the untrained hand did not contribute to the improved perception of force generation after unilateral exercise with the opposite limb. Thus, the improved accuracy with the untrained hand is dependent upon transfer from the ‘trained’ to the ‘untrained’ M1 and/or a concurrent generation and recalibration of the copy in the untrained M1.

Many other mechanisms have been postulated to mediate the contralateral effects of a unilateral exercise [19]. TMS studies have demonstrated activation within the non-exercised M1 and increased excitability of the corticospinal pathway after contralateral unilateral isometric exercise [49]–[51]. A recent study from Hortobagyi et al. [16] investigated whether cross education produced by isometric unimanual exercise was mediated by increases in corticospinal excitability of the non-exercised M1 or a reduced inter-hemispheric inhibition (IHI) from the trained to the non-trained hemisphere. After 1,000 contractions of the right first dorsal interosseus (FDI) at 80% MVC performed over 20 sessions, significant strength gains were evident in the right (50%) and left (28%) FDI muscle. The strength gains were accompanied by a 6% increase in corticospinal excitability of the non-exercised M1, however, this result did not correlate with the observed cross education. IHI decreased progressively during the training phase and after 20 sessions had fallen by 31%, a result that correlated strongly with the observed cross education. Moreover, Camus et al. [21] also found a reduced IHI from an ‘untrained’ M1 following a pinch task. Thus, it seems that attenuation of IHI contributes to interlimb transfer and the ability to produce maximal force by the untrained homologous muscle after unimanual exercise [16], [52]. Although IHI was not measured in the present study, given recent research, it seems plausible to suggest that the transfer of learning evidenced after the exercise alone may have been the result of a decreased IHI, allowing for a bilateral increase in the excitability of each M1 *during* the task.

### Rating of Perceived Exertion

An increased RPE demonstrates an increase in the sense of exertion after an intervention; whereas, a lack of change in RPE demonstrates no discernible difference in the sense of exertion. The associated transfer of learning to the untrained hand, evidenced through a decreased error in force output production, was accompanied without an increased RPE in contrast to the trained hand, suggestive of a lower sensation of fatigue (Figure 3). Indeed, the RPE in the trained hand would have increased due to the nature of the exercise; however, this increased sense of effort may have itself disrupted the ability to accurately determine pinch force post-exercise in both hands [34]–[36]. Previous investigations have measured EMG and the sense of effort in an attempt to establish if the two are related. During sustained low-force contractions, the sense of effort has been shown to rise well beyond increases in EMG, demonstrating they are poorly related [53]. In a non-fatigued state, EMG that equates to ‘mild’ effort equates to ‘large’ effort (in terms of RPE) when fatigued [53]. An explanation for this mismatch during fatiguing exercise, is that motoneurons require more descending input to drive them to the same output [54]. The sense of effort is unlikely to derive from a direct corollary of motor cortical output cells and the same reasoning may be applied to corticospinal neurons [55]. Small-diameter muscle afferents can exert complex effects on the excitability of motoneurons; although they are known to facilitate some motoneuron pools, they are known to directly inhibit others [56]. At the level of the cortex, small-diameter afferents act to impair maximal voluntary drive and can decrease responses to motor cortical stimulation [57]. Thus, the heightened sensation of fatigue in the trained hand and the firing of small diameter muscle afferents, might have contributed to the lack of learning and the ability to accurately determine force output post-exercise in the trained hand. Our data suggest that the sensation of fatigue is specific to the trained limb only and does not impair the transfer of learning to the untrained limb.

### Corticospinal Excitability

In what might be viewed as a limitation of the present study, but in line with previous research, no changes were seen in MEP amplitude in either hand [26] despite rTMS disrupting the transfer of learning when combined with the exercise task. Muellbacher et al. [26] also reported a lack of altered excitability in the trained hand and reasoned that interactions between learning-related excitatory effects and rTMS-related inhibitory effects are responsible. Low frequency rTMS is expected to result in a reduced corticospinal excitability [58]. However, scrutiny of the literature shows a diverse response to low frequency rTMS. Some investigations have found considerable inter-individual variation in response to rTMS [59], [60], others have found an inhibited MEP response [61]–[63], whilst some, as in the present study, have found no effect [14], [64]–[66]. That rTMS did not modify corticospinal excitability at rest in the present study, may have been due to the stimulation intensity being set at 90% of rMT [67]. There might be a different susceptibility of motor neurons that did not respond to rTMS delivered at 90% rMT compared to the interneurons that serve for the transfer of learning. Similarly, Muellbacher et al. [26] also report unchanged corticospinal excitability after a period of rTMS when using 115% of rMT as the chosen stimulation intensity. Thus, the lack of change in MEPs after any intervention in the present study (Figure 5) may have been a result of the chosen stimulus intensity. Suzuki et al. [68] recently investigated reciprocal changes in input-output curves of MEPs while learning motor skills. Their data demonstrate that the relationship between MEP amplitude (in the extensor and flexor carpi radialis muscles) and stimulus intensity reaches a plateau at stimulus intensities of approximately 140–150% rMT. Thus, the selected stimulation intensity in the present study (140% rMT), which was based on previous research also showing no change in MEPs [26], might have saturated the neural network and prevented any changes in corticospinal excitability to be exposed. Nevertheless, the important finding from the present study was that the rTMS protocol disrupted the exercise-induced transfer of learning, which is evident with unilateral isometric exercise.

### Conclusion

In conclusion, these data are consistent with the evidence showing that the ‘active’ M1 contributes to the transfer of learning after a period of unilateral isometric exercise. rTMS blocked transfer of force perception and the heightened sensation of fatigue in the trained hand attenuated improvements in the ability to accurately perceive force output. The present results may have clinical relevance to neurological and orthopedic patients who could use unilateral exercise as a method to help offset the negative effects of an immobilised contralateral limb.
